# Secure Nearest Neighbor Query on Crowd-Sensing Data

**DOI:** 10.3390/s16101545

**Published:** 2016-09-22

**Authors:** Ke Cheng, Liangmin Wang, Hong Zhong

**Affiliations:** 1 School of Computer Science and Technology, Anhui University, Hefei 230601, China; chengke@ahu.edu.cn; 2 School of Computer Science and Communication Engineering, Jiangsu University, Zhenjiang 212013, China; wanglm@ujs.edu.cn

**Keywords:** secure nearest neighbor, crowd-sensing, privacy-preservation, secure two-party computation, collusion attack

## Abstract

Nearest neighbor queries are fundamental in location-based services, and secure nearest neighbor queries mainly focus on how to securely and quickly retrieve the nearest neighbor in the outsourced cloud server. However, the previous big data system structure has changed because of the crowd-sensing data. On the one hand, sensing data terminals as the data owner are numerous and mistrustful, while, on the other hand, in most cases, the terminals find it difficult to finish many safety operation due to computation and storage capability constraints. In light of they Multi Owners and Multi Users (MOMU) situation in the crowd-sensing data cloud environment, this paper presents a secure nearest neighbor query scheme based on the proxy server architecture, which is constructed by protocols of secure two-party computation and secure Voronoi diagram algorithm. It not only preserves the data confidentiality and query privacy but also effectively resists the collusion between the cloud server and the data owners or users. Finally, extensive theoretical and experimental evaluations are presented to show that our proposed scheme achieves a superior balance between the security and query performance compared to other schemes.

## 1. Introduction

Along with the popularization of mobile Internet and Internet of Things, a large quantity of ordinary users and sensor nodes have become involved in the perception and collection activities around the state of the environment. Hence, brand-new crowd sensing data emerge as the times require, and researchers are beginning to be concerned about the influence of such data on human life [1,2,3,4,5], including medical treatment, social networks, environmental monitoring, transportation, etc. The sensor data may contain private user details, especially for sensors that can collect location coordinates for Location Based Service (LBS). The cloud party has brought vast amounts of sensitive data together after data owners outsource their databases to the cloud server provider. Therefore, the inappropriate use of crowd sensing data, which not only contain user locations but also personality habits, health condition, social status and other sensitive information, brings great challenges to data confidentiality and user privacy [6,7,8].

**Figure 1:**
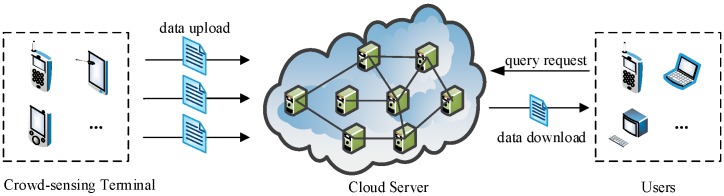
Multi Owners and Multi Users (MOMU) structure based upon crowd sensing data.

**Figure 2:**
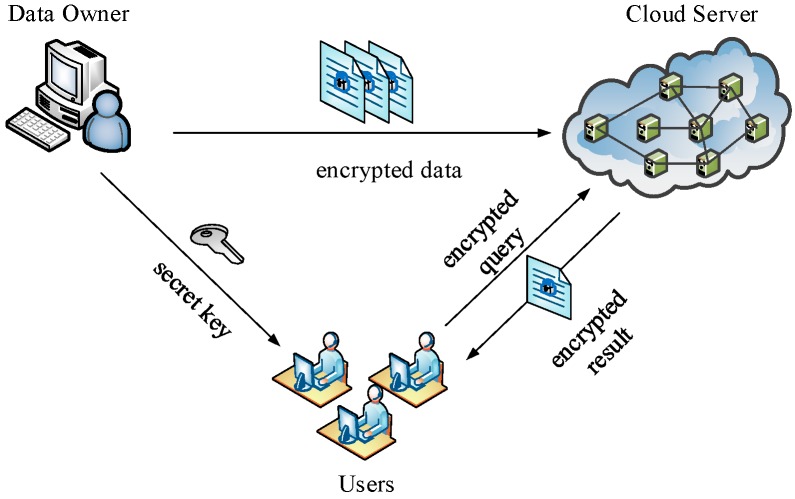
Traditional (Single Owner and Multi Users) SOMU model.

To protect the confidentiality of the location data in the cloud, one straightforward way is to encrypt data by the data owner (Owner) before outsourcing. In addition, to preserve user privacy, authorized users (Users) need to perform a complex series of encryption and decryption operations during query execution. However, this approach cannot be directly applicable to crowd sensing data because the mobile terminals in crowd sensing networks fail to perform the current big computation limited to compute and storage capability. More importantly, mobile terminals, which are the source of crowd sensing data, are mutually-distrusting as data owners. This situation makes up a totally different service structure, as depicted in Figure 1. We call it Multi Owners and Multi Users (MOMU) cloud services structure based upon crowd sensing data, referred to as MOMU structure. It is different from the traditional Single Owner and Multi Users (SOMU) structure portrayed in Figure 2, in which only one data owner has a large number of data and outsources them to the cloud, then authorized users access those data for issuing queries.

In this paper, we focus on the secure nearest neighbor (SNN) problem on crowd-sensing location data (MOMU structure is a typical structure in the applications of crowd sensing [9,10]), since LBS is the current hot topic in the study of big data [11,12,13], furthermore, nearest neighbor (NN) queries are fundamental in LBS [14,15]. In the past few years, researchers have proposed various methods [15,16,17,18,19] to address the SNN problem in SOMU model. The work in [16] uses a new encryption scheme (ASPE) to preserve scalar product between the query vector and any vector for distance comparison, which is sufficient to find NN. Hu et al. [17] propose a solution based on privacy homomorphism encryption scheme (ASM-PH). Instead of finding exact NN, [15] allows a cloud party to approximate it based on secure Voronoi diagram (SVD). Similar to [15], the work in [18] also uses Voronoi to raise efficiency. Elmehdwi et al. [19] propose a novel protocol over encrypted data based on Paillier cryptosystem [20], which can calculate encrypted distance between data record and query record in a secure way.

One important observation about these prior works is that the data owners are all assumed to be a single trusted party. Hence, in the MOMU structure, it is impractical to share the secret key between all the data owners and users just like existing solutions [15,16,17,18,19] because the compromise of any data owner would be a threat to data security of other owners. For instance, in a cloud system based on key-sharing, if an owner colludes with the cloud, the other owners’ data stored in cloud will be leaked because they could be decrypted with a sharing key. A natural idea is that multiple data owners could use their own unique keys. However, the SNN query across the data encrypted by different keys is another challenge (e.g., data availability, key management, etc.). In addition, the mobile terminals in crowd sensing networks cannot fulfill the requirements for computation and storage capability of the end-user in traditional methods. Therefore, the methods based on SOMU structure cannot be applied to crowd-sensing cloud server directly.

To address those challenges, our insight is that there is generally a proxy server of service providers in a cloud environment. Thus, we can use the proxy server to share the hard work for the end-user. In order to ensure availability of encrypted data by different keys, we also provide a series of protocols of secure two-party computation coordinating to the proxy architecture, which not only protects the confidentiality of the location data from various data owners but also allows the specified user to perform the SNN query efficiently. In summary, our paper makes the following contributions:

- We propose a Security Architecture over MOMU Cloud Service System (SAMOMU) model based on partition of public cloud and proxy server to meet the security and performance requirements of MOMU structure.
- In the SAMOMU model, a method to solve the SNN problem is presented by combining SVD method and a series of secure two-party computation protocols.
- We present an extensive experimental evaluation of the proposed scheme, which shows that the proposed method has good performance for crowd-sensing data.

The remainder of this paper proceeds as follows. Related works are surveyed in Section 2. We define our system model and design goals in Section 3. A set of basic security protocols which are utilized in our scheme are provided in Section 4. Section 5 presents the details of our scheme. The security and performance analysis are carried out in Section 6. Finally, Section 7 concludes the paper and discusses potential future directions.

## 2. Related Works

In this section, we first review several nearest neighbor query methods for location privacy in LBS, and then we present an overview of the existing SNN techniques.

### 2.1. Query Location Privacy in LBS

In traditional LBS model, the methods should ensure location privacy in the sense that the user does not reveal any information about his location to the LBS provider. In this case, LBS server acts as the role of data owner. As a consequence, there is a simpler security requirement compared with the SNN query in the cloud, which focuses mainly on privacy preserving for the users.

In general, several main techniques for location privacy have been investigated in current studies. The first is the cloaking regions method [21,22], which assumes a trust anonymous party between the user and the server for transforming actual locations into vague locations. Obviously, the anonymizer becomes a communication bottleneck and a vulnerable point of attack. To count this privacy attack, Gao et al. [23] propose a distributed structure for location privacy protection without a centralized anonymous server. Another category of work relies on Private Information Retrieval (PIR) [24] to provide strong location privacy. This technique allows users to retrieve an object stored by a server without revealing which record he is retrieving. However, these PIR-based solutions [25,26] are still not efficient enough to be implemented on a real system.

### 2.2. Existing SNN Techniques

Existing SNN techniques generally rely on SOMU model, which only contains a single trusted data owner, as depicted in Figure 2. Compared to the MOMU model, the significant difference is: the MOMU model involves multiple mutually-distrusting data owners.

In the methods [15,16,17,18,19] based on SOMU model, the data owner outsources his database and DBMS functionalities (e.g., NN query) to the cloud server providers where only trusted users are allowed to query the host data. Wong et al. [16] proposed a new encryption scheme (ASPE) that preserves the relative distances of all the database point to any query point that is sufficient to find NN. ASPE transforms data points and queries with secret matrices, which are symmetric keys for the encryption scheme. Thus, it must be shared with both the data owner and query users. As an alternate, Hu et al. [17] proposed a method based on Privacy Homomorphism (ASM-PH) encryption scheme. During query processing, data owner sends the encrypted shadow index to user, and user needs to traverse the index locally to compute the distance between query point and an indexed point with the help of server. However, the methods in [16,17] are not secure because they are prone to chosen-plaintext attacks [15].

To further improve the query performance, Yao et al. [15] designed a novel SNN method based on secure Voronoi diagram (SVD). Instead of return exact NN, they allow a cloud server to return a relevant data partition. What is more notable, is that the work in [18] also used Voronoi and order-preserving encryption (OPE) to solve the SNN problem accurately. Although it can provide exact result, the solution incurs expensive overhead of computation and communication on the end-user. More importantly, the encryption schemes used in [15,18] are symmetric, and both the data owners and users have to share the secret key, which make it impractical in MOMU structure where there are multiple mutually-distrusting data owners.

Recently, Elmehdwi et al. [19] proposed a number of novel protocols over encrypted data based on Paillier cryptosystem [20], which can further increase security during query execution. They assume the existence of two semi-honest cloud servers *P*_1_ and *P*_2_ such that the encrypted data is known only to *P*_1_, whereas the secret key is just revealed to *P*_2_. Using the secure protocols, *P*_1_ collaborate with *P*_2_ for the final result after receiving an encrypted query from the user. However these protocols cannot be put into use for inefficiency.

Crowd-sensing cloud server is based on the MOMU structure, in which the number of data owners increases and computing power of end-users decreases compared with SOMU structure. These changes about objective conditions cause the changes of the security and performance requirements. Hence, the methods above do not apply to MOMU structure in which there are multiple mutually-distrusting and the end-user cannot afford huge costs for compute or storage.

## 3. System Model and Design Goals

In this section, we formalize the system model, security and privacy requirements, and describe our design goals.

### 3.1. System Model

**Figure 3:**
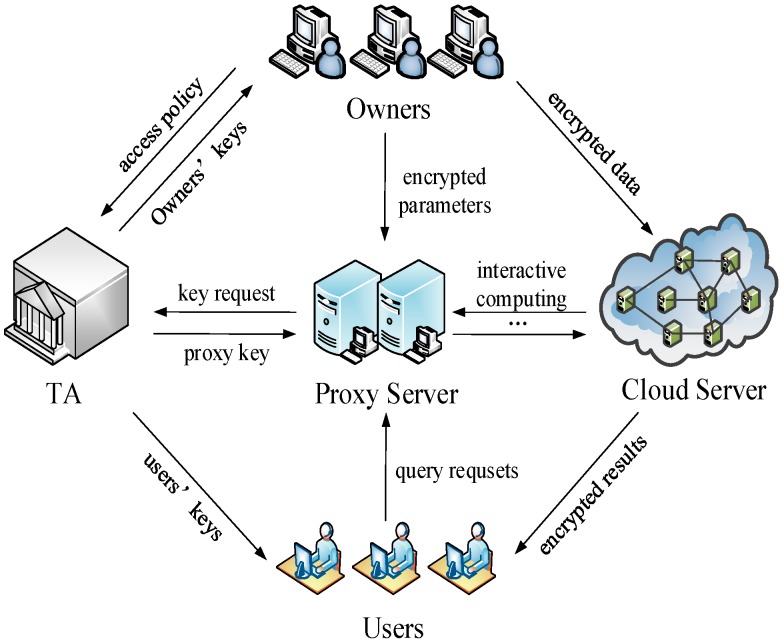
Security Architecture over MOMU Cloud Service System (SAMOMU).

The cloud service system based upon crowd-sensing data is actually aggregations of the crowd-sensing system and the cloud system. The terminals in this system are divided into two kinds of entities in the function: the data owner (Owner) and the data user (User). As a data owner, the terminal will outsource his/her data to the cloud for efficient storage and management. In fact, there is generally a proxy server of service providers in a cloud service based on crowd-sensing data. With the crowd-sensing data in VANET, for example, the data collected through VANET are uploaded to the cloud and governed by the traffic administrative department while the users such as automobile manufacturers, garages and insurance companies need to access the relevant data. Nowadays, large companies usually set up their own proxy server for different types of server. In this scenario, the traffic administrative department can be viewed as a trusted authority (TA). When a user wants to check the information about insurance and vehicle maintenance, he has to access them using the proxy of the insurance company and the garage, respectively. This is similar to the social network, which may contain a variety of services in regard to foods, sports, garments and so on, a user acquires different kinds of data through the corresponding proxy servers of service providers. To this end, we propose a Security Architecture over MOMU Cloud Service System (SAMOMU), as depicted in Figure 3.

Our system consists of five types of generic entities: Data Owners (Owners), Data Users (Users), Cloud Server (CS), Proxy Server (PS) and Trusted Authority (TA).

(1) Data Owners (Owners): We assume that our system requires *m* Owners, which are generally played by the sensor nodes, mobile phones and vehicle terminals, denoted as Own = {*O*_1_, *O*_2_, …, *O_m_*}. Data are generated by Owners, encrypted using their secret key and then outsourced to CS for storage.
(2) Data Users (Users): The system assumes there are *n* Users with limited computation and communication resources, denoted as Usr = {*U*_1_, *U*_2_, …, *U_n_*}. Note that the terminals that act as Owners could also take up positions as Users. They forward queries to the CS via the PS for the nearest neighbor.
(3) Cloud Server (CS): The CS stores all the encrypted data outsourced from Owners. After received NN requests from the PS, the CS interacts with the PS to process the data and returns the results to the Users.
(4) Proxy Server (PS): The PS takes on the task of providing those users with proxy services. In reality, much useful information is distributed among the crowd sensing networks, hence the PS normally caches the parsing results or extracts the metrics of interest. In SAMOMU model, the PS will host part of the computing task for Users.
(5) Trusted Authority (TA): TA is assumed to be trusted by all the other entities in the system to distribute and manage all the private keys, and to generate some parameters involved in the system.

Note that our systems are scalable and efficient for users. Specifically, users do not need to know the identities of other users or the total number of users involved in computation. Most importantly, because of the PS, the computation is non-interactive to users—users only need to outsource encrypted data initially and remain offline until retrieving encrypted outputs. It has been proven that the traditional single server model for secure outsourced computation cannot completely eliminate interactions between the user side and the server side (due to the impossibility of program obfuscation). The defect of this architecture is that the PS is likely to become a Single Point of Failure (SOF). However, in the real world, all service providers have the separated proxy server, which is totally independent of each other. Furthermore, service providers can adopt the hot-standby technique for solving the SOF from a view of engineering. Although the providers need to increase investment in infrastructure, it would make for a pleasant user experience in return. This is also the basic motivation of the paper.

### 3.2. Security and Privacy Requirements

In our security and privacy model, we assume PS and CS are both semi-honest (i.e., honest-but-curious). Meanwhile, we also assume these two servers are non-colluding. It means that neither of these two servers intends to corrupt users’ data or computation process to prevent users from utilizing data correctly, but each server will try to learn the content of users’ data (i.e., inputs), intermediate or final results of the computation without colluding with another server.

We remark that those assumptions are not initiated by our work, but rather derive from the related research [19,27,28,29]. According to the requirements of crowd-sensing scenario, the SAMOMU partitions server functions under the management of the TA. Actually, the security of our system is stronger than the Two-Clouds architecture [19], because the TA would be charge of the key management, the collusion between the PS and the CS cannot breakdown the full security of our system. To provide a flexible tradeoff between security and performance, we define the concrete data confidentiality and query privacy to against adversary *Adv* as follows.

**Definition** **1 (Data Confidentiality Definition).** *Upon completion of the SAMOMU model, Adv cannot learn any plain data stored in the CS when Adv did not collude with any Owners. If an Owner was captured by Adv, the adversary would not get any assistance to obtain sensor data generated* *by other Owners.*

**Definition** **2 (Query Privacy Definition).** *Neither the query point nor the result for* *users should be reveal to the Adv.*

To satisfy these privacy requirements, the active adversary *Adv* in our model has the following attacking abilities: *Adv* may eavesdrop all the communication links to get the encrypted data. In addition, *Adv* may compromise CS, some Users and Owners simultaneously, but subjects to the following restrictions: (1) *Adv* cannot compromise the CS and the PS at the same time; and (2) in a process of query, *Adv* cannot compromise the User who launched this query. Moreover, we do not aim to protect access pattern in this paper due to the extremely high complexity, i.e., to protect it, the algorithm has to “touch” the whole dataset [24].

### 3.3. Design Goals

In order to achieve the SNN query under SAMOMU model, our method will fulfill privacy and performance guarantees as follows:

- Data confidentiality and query privacy: The data confidentiality and query privacy as described in the Definitions 1 and 2 should be guaranteed.
- Reduce the end-users’ cost: The end-users in SAMOMU model generally have limited computation and communication resources, thus our method should be designed for reducing the end-users’ cost by using the PS efficiently.
- Access Control: A large number of parties are involved in the system, therefore control of the user’s access request by attribute-based encryption (ABE) [30] is necessary.

We list the main technologies used in our method in Table 1; these cannot apply to our method directly, and the improvements and combinations of them are technical contribution of our work.

## 4. Basic Security Protocols

In this section, we present a set of secure two-party computation protocols that will be used as sub-routines while constructing our proposed scheme in Section 5. We firstly introduce an encryption scheme using secret-sharing [31], based here to build our protocols.

### 4.1. The Encryption Scheme Based on Secret-Sharing

Under secret sharing, the encryption scheme used in [31] aims to split a plaintext into a secret key and a ciphertext for data confidentiality. The concrete algorithm is showed in Definition 3.

**Definition** **3.** *The secret sharing encryption process consists of* *two steps:*

***Step 1 (Key Generation).*** *Generate a public parameter PP = <g, n> in the follow way: choose randomly two prime numbers p and q, then compute n = p × q,* φ(n) *= (p − 1)·(q − 1). Choose randomly a positive number g that is co-prime with n. Generate randomly a secret key sk = {m, a} (0 < m, a < n).*

***Step 2(Share Computation).*** *Given a sensitive value x, choose randomly a number r, the encrypted value E_sk,r_(x) is given by E_sk,r_(x) = x·(mg^ra^ mod n)^−1^ mod n, where ( )^−1^ denotes the modular inversion. To recover x, one needs all shares sk, r and E_sk__,r_(x) and compute D_sk__,r_(E_sk__,r_(x)) = E_sk__,r_(x)·(mg^ra^ mod n) mod n. We refer the reader to [31] for correctness and security proof of this scheme.*

### 4.2. Secure Two-Party Computation Protocols

We present a set of protocols based on the encryption scheme above. All of the below protocols are considered under two-party semi-honest setting: Data Normalization (DataNorm) protocol, Secure Distance (SecDist) protocol, Secure Compare (SecComp) protocol, Secure Minimum of *k* Numbers (SecMin*_k_*) protocol.

**Data Normalization (DataNorm).** We assume that a party *P*_1_ holds a secret key *sk*_1_ = {*m*_1_, *a*_1_}, a random number *r*_1_, a target key *sk*_2_ = {*m*_2_, *a*_2_} and a target number *r*_2_ while a party *P*_2_ has encrypted value $E_{sk_{1},r_{1}}(x)$. The goal of the DataNorm protocol is to compute the encryption of *x*, which is encrypted by *sk*_2_ and *r*_2_. At the end, the output is known only to *P*_2_. In our query scheme described in Section 5, we will use the DataNorm protocol to make a data normalization over the encrypted data, although those data were encrypted using different keys of multiple data owners. Thanks to this, we can ensure availability of encrypted data. The protocol is shown in Algorithm 1.

**Algorithm 1.** DataNorm ($E_{sk_{1},r_{1}}(x)$, {*sk*_1_,*r*_1_}, {*sk*_2_,*r*_2_}) − $E_{sk_{2},r_{2}}(x)$Require: *P*_1_ has *sk*_1_ = {*m*_1_, *a*_1_}, *sk*_2_ = {*m*_2_, *a*_2_},*r*_1_, *r*_2_; *P*_2_ has $E_{sk_{1},r_{1}}(x)$
(1)*P*_1_:
(a)Pick two random numbers *m*_3_, *a*_3_, *r*_3_(b)*p* ← *a*_3_^−1^(*r*_2_*a*_2_ − *r*_1_*a*_1_) mod φ(n)(c)*q* ← *m*_1_*m*_3_*^p^m*_2_^−1^ mod *n*(d)*s* ← ($m_{3}g^{a_{3}}$ mod *n*)^−1^(e)Send *p*, *q*, *s* to *P*_2_(2)*P*_2_:
(a)$E_{sk_{2},\;r_{2}}(x)$ ← $E_{sk_{1},\;r_{1}}(x)$·*q*·*s^p^*

**Definition** **4 (Correctness)**. *If DataNorm protocol presented in Algorithm 1 is correct, a party P_2_ can get the encryption of x, which is encrypted by* *sk_2_ and r_2_.*

**Proofs of Correctness**.  We can use *sk*_2_ and *r*_2_ to decrypt ciphertext of *P*_2_, converting from $E_{sk_{2},r_{2}}(x)$ back to plain text *x*. The process is as follows.

$$\begin{array}{ll} D_{sk_{2},\;r_{2}}(E_{sk_{2},\;r_{2}}(x)) & =E_{sk_{1},\;r_{1}}(x)\cdot q\cdot s^{p}\cdot m_{2}\cdot g^{r_{2}a_{2}}\\ & =E_{sk_{1},\;r_{1}}(x)\cdot m_{1}\cdot {m_{3}}^{p}\cdot {m_{2}}^{-1}\cdot s^{p}\cdot m_{2}\cdot g^{r_{2}a_{2}}\\ & =E_{sk_{1},\;r_{1}}(x)\cdot m_{1}\cdot {m_{3}}^{p}\cdot {\left({\left(m_{3}\cdot g^{a_{3}}\right)}^{-1}\right)}^{p}\cdot g^{r_{2}a_{2}}\\ & =E_{sk_{1},\;r_{1}}(x)\cdot m_{1}\cdot {\left(g^{a_{3}\cdot ({a_{3}}^{-1}(r_{2}a_{2}-r_{1}a_{1}))}\right)}^{-1}\cdot g^{r_{2}a_{2}}\\ & =E_{sk_{1},\;r_{1}}(x)\cdot m_{1}\cdot g^{r_{1}a_{1}}=x \end{array}$$

□

**Secure Distance (SecDist).** Consider a party *P*_1_ with secret key *sk*, a secret share *r* and a party *P*_2_ with private input *E_sk_*_,*r*_(***X***), *E_sk_*_,*r*_(***Y***). Here, ***X*** and ***Y*** are two-dimensional vectors where *E_sk_*_,*r*_(***X***) ≤ *E_sk_*_,*r*_(*x*_1_), *E_sk_*_,*r*_(*x*_2_)>, and *E_sk_*_,*r*_(***Y***) = <*E_sk_*_,*r*_(*y*_1_), *E_sk_*_,*r*_(*y*_2_)>. The goal of the SecDist protocol is to compute *E_sk_*_,*r*_(${|X-Y|}^{2}$), where ${|X-Y|}^{2}$ denotes the Euclidean distance between *X* and *Y*. During this protocol, no information regarding *X* and *Y* is revealed to *P*_1_ and *P*_2_. The SecDist protocol described in Algorithm 2 will be used as a sub-routine to construct our SNN method in Section 5. 

**Algorithm 2.** SecDist(*E_sk_*_,*r*_(*X*),*E_sk_*_,*r*_(*Y*)) − $E_{sk,r}({|X-Y|}^{2})$Require: *P*_1_ has *sk* = {*m*, *a*}, *r*; *P*_2_ has $E_{sk,r}({|X-Y|}^{2})$
(1)*P*_2_:
(a)$E_{sk^{\prime },r}({|X-Y|}^{2})$ ← (*E_sk_*_,*r*_(*x*_1_) − *E_sk_*_,*r*_(*y*_1_))^2^ + (*E_sk_*_,*r*_(*x*_2_) − *E_sk_*_,*r*_(*y*_2_))^2^(2)*P*_1_:
(a)$m^{\prime }$ ← *m*^2^, $a^{\prime }$ ← 2*a*, $sk^{\prime }$ ← {$m^{\prime }$,$a^{\prime }$}(3)*P*_1_ and *P*_2_:
(a)$E_{sk,r}({|X-Y|}^{2})$ ← DataNorm($E_{sk^{\prime },r}({|X-Y|}^{2})$,{$sk^{\prime }$, *r*},{*sk*, *r*})(b)*P*_2_ get $E_{sk,r}({|X-Y|}^{2})$

**Definition** **5 (Correctness)**. *If SecDist protocol presented in Algorithm 2 is correct, a party P_2_ can get the value* $E_{sk,r}({|X-Y|}^{2})$, *which can be decrypted by sk and r.*

**Proofs** **of Correctness**. We can use *sk* and *r* to decrypt ciphertext of *P*_2_, converting from $E_{sk,r}({|X-Y|}^{2})$ back to plain text ${|X-Y|}^{2}$. The process is as follows.

$$\begin{array}{ll} D_{sk,r}(E_{sk,r}(|{X-Y|}^{2})) & =D_{sk^{\prime },r}(E_{sk^{\prime },r}(|{X-Y|}^{2}))\\ & =D_{sk^{\prime },r}({\left(x_{1}\cdot {\left(m\cdot g^{ra}\right)}^{-1}-y_{1}\cdot {\left(m\cdot g^{ra}\right)}^{-1}\right)}^{2}\\ & \;+{\left(x_{2}\cdot {\left(m\cdot g^{ra}\right)}^{-1}-y_{2}\cdot {\left(m\cdot g^{ra}\right)}^{-1}\right)}^{2})\\ & =D_{sk^{\prime },r}(({\left(x_{1}-y_{1}\right)}^{2}+{\left(x_{2}-y_{2}\right)}^{2})\cdot {\left(m^{2}\cdot g^{2ra}\right)}^{-1})\\ & =({\left(x_{1}-y_{1}\right)}^{2}+{\left(x_{2}-y_{2}\right)}^{2})\cdot {\left(m^{2}\cdot g^{2ra}\right)}^{-1}\cdot m^{2}\cdot g^{2ra}\\ & ={\left(x_{1}-y_{1}\right)}^{2}+{\left(x_{2}-y_{2}\right)}^{2} \end{array}$$

□

**Secure Compare (SecComp).** In this protocol, *P*_1_ holds *sk* = {*m*, *a*}, *r* and *P*_2_ holds *E_sk_*_,*r*_(*x*), *E_sk_*_,*r*_(*y*). The goal of the SecComp protocol is to compare *x* with *y* without revealing any information about *x* and *y* to *P*_1_ and *P*_2_. This protocol returns true if *x* > *y*, otherwise it returns false. The protocol is shown in Algorithm 3.

**Algorithm 3.** SecComp(*E_sk_*_,*r*_(*x*), *E_sk,r_*(*y*)) → true/false.Require: *P*_1_ has *sk* = {*m*, *a*}, *r;P*_2_ has *E_sk_*_,*r*_(*x*) and *E_sk,r_*(*y*)
(1)*P*_1_:
(a)Pick a random positive number *z*(b)*E_sk_*_,*r*_(*z*) ← *z*·*m*·*g^ra^*(c)Send *E_sk,r_*(*z*) to *P*_2_(2)*P*_2_:
(a)*t* ← (*E_sk_*_,*r*_(*x*) − *E_sk_*_,*r*_(*y*))·*E_sk_*_,*r*_(*z*)(3)*P*_1_:
(a)$sk^{\prime }$ = 〈*m*^2^, 2*a*〉,$sk^{\prime \prime }$ = 〈1, 0〉(4)*P*_1_ and *P*_2_:
(a)*f* ← DataNorm(*t*,$sk^{\prime }$,$sk^{\prime \prime }$)(5)*P*_2_:
(a)**IF** *f* > 0 **THEN** **RETURN** true**ELSE** **RETURN** false **RETURN** false

**Definition** **6 (Correctness)**. *If SecComp protocol presented in Algorithm 3 is correct, a party P_2_ will get* *a f > 0 iff x > y.*

**Proofs** **of Correctness.** *f* = $E_{sk^{\prime \prime },r}((x-y)\cdot z)$ = (*x* − *y*)·*z*·1·*g^r^*^·0^ = (*x* − *y*)·*z* and *z* > 0, so iff *f* > 0 then *x* > *y*, else *x* < *y*. □

**Figure 4:**
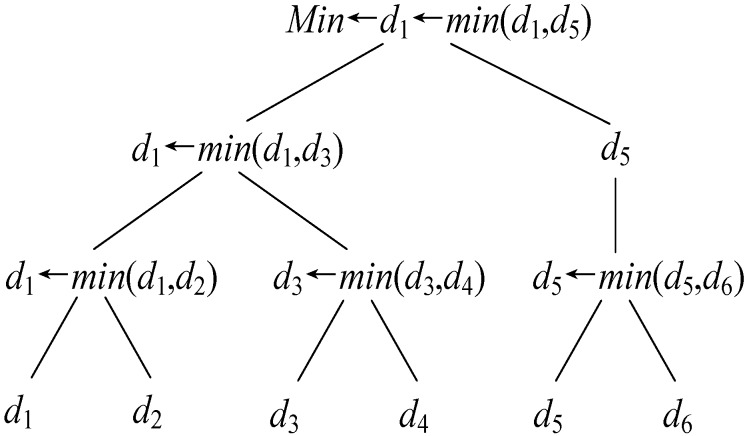
The minimum value solving process by SecMin*_k_* for *k* = 6.

**Secure Minimum of *k* Numbers (SecMin*_k_*).** We assume that *P*_1_ has *sk* = {*m*, *a*}, *r* and *P*_2_ has *E_sk_*_,*r*_(*x*_1_), *E_sk_*_,*r*_(*x*_2_), …, *E_sk_*_,*r*_(*x_k_*), the goal of the SecMin*_k_* protocol is to securely compute *Min* = min(*x*_1_, *x*_2_, …, *x_k_*). During this protocol, no information regarding *x_i_* (1 ≤ *i* ≤ *k*) is revealed to *P*_1_ and *P*_2_. On the basis of the SecComp protocol, all the values in the SecMin*_k_* protocol are compared in pairs using the divide-and-conquer strategy. Note that the computation complexity of SecMin*_k_* is bounded by *O*(log_2_*k*). For instance, *P*_1_ has *sk* = {*m*, *a*}, *r* and *P*_2_ has *E_sk_*_,*r*_(*x*_1_), *E_sk_*_,*r*_(*x*_2_), …, *E_sk_*_,*r*_(*x*_6_), the minimum value solving process is present in Figure 4. The protocol is shown in Algorithm 4.

**Algorithm 4.** SecMin*_k_*(*E_sk_*_,*r*_(*x*_1_), *E_sk_*_,*r*_(*x*_2_), …, *E_sk_*_,*r*_(*x_k_*)) → *Min.*Require: *P*_1_ has *sk* = {*m*, *a*}, *r;*
*P*_2_ has *E_sk_*_,*r*_(*x*_1_), *E_sk,r_*(*x*_2_), …, *E_sk_*_,*r*_(*x_k_*)
(1)*P*_2_:
(a)*d_i_* ← *E_sk_*_,*r*_(*x_i_*), FOR 1 ≤ *i* ≤ *k*(b)*num* ← *k*(2)*P*_1_ and *P*_2_, **FOR** *i* = 1 **TO** $\left\lceil \log_{2}k\right\rceil$:
(a)**FOR** 1≤j≤⌊num/2⌋: **IF** *i* = 1 **THEN**   **IF** SecComp(*d*_2*j*−1_, *d*_2*j*_) **THEN** *d*_2*j*−1_ ← *d*_2*j*_ **ELSE**   IF SecComp(*d*_2*i*(*j*−1)+1_, *d*_2*ij*−1_) **THEN** *d*_2*i*(*j*−1)+1_ ← *d*_2*ij*−1_(b)num←⌈num/2⌉(3)*P*_1_:
(a)*Min* ← *d*_1_

## 5. The Proposed SNN-SAMOMU Query Scheme

**Figure 5:**
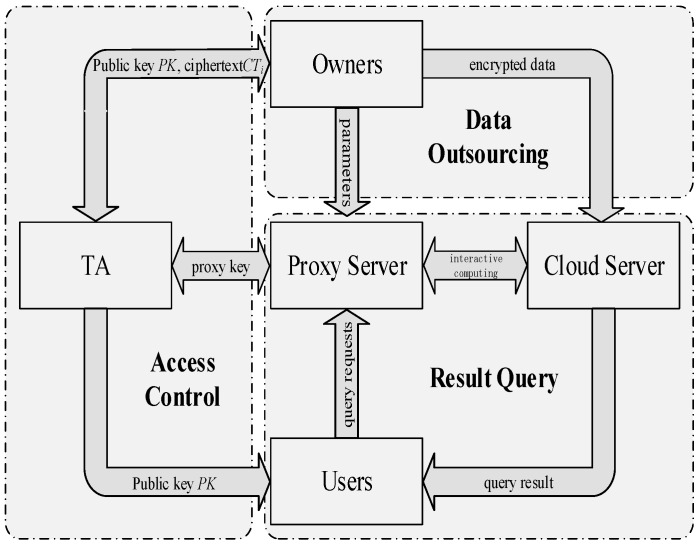
The query framework based on SNN-SAMOMU model.

Based on the secure two-party computation protocols presented in Section 4, we propose a SNN query scheme in SAMOMU model, which consists of the following phases: System Setup, Data Outsourcing, Access Control and Result Query. Figure 5 shows a SNN-SAMOMU query framework. Firstly, TA initializes the system, then data owners encrypt their data and outsource the corresponding encrypted data to CS while uploading random parameters to PS. To guarantee the access control, data owners use attribute-based encryption (ABE) to encrypt their own secret keys and send them to TA for management. Once the data user is authenticated by TA, PS will receive a proxy key from TA for computation. In the result query phase, PS will cooperate with CS to perform a query protocol for a result point as output to the user. Finally, we present two strategies to boost performance of our scheme.

### 5.1. System Setup

The TA calls the Key Generation algorithm to generate a public parameter *PP*, the users’ keys for *m* Users and the owners’ keys for *n* Owners. Let *Key_O_i_* (1 ≤ *i* ≤ *m*) and *Key_U_j_* (1 ≤ *j* ≤ *n*) denote users’ keys and owners’ keys, respectively. The TA publishes *PP* and sends the keys to the corresponding Owners and Users via secure channels.

### 5.2. Data Outsourcing

The Owners divide the data space into *K* disjoint intervals through SVD algorithm [15] locally, then use the *PP* and owners’ keys to encrypt their own data and index by the encryption scheme described in Section 4.1. Finally, the encrypted data and index are outsourced to the CS. Our data outsourcing protocol runs in the following four steps.

**Figure 6:**
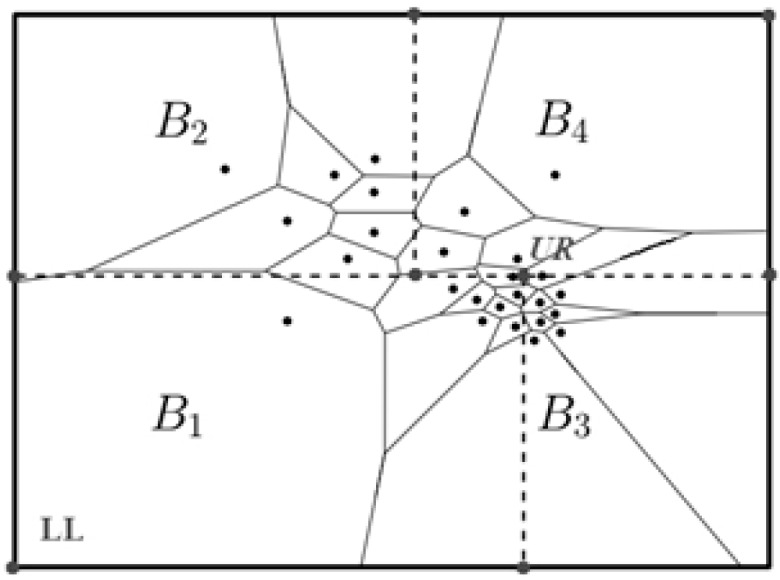
The secure Voronoi diagram (SVD) method for *K* = 4.

**Figure 7:**
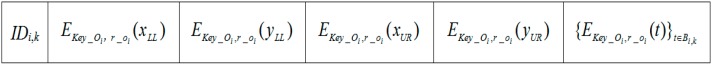
Format of outsourced data.

(1) The data owner *O_i_* receives a public parameter *PP* and his key *Key_O_i_*.
(2) *O_i_* divides the data space, which is corresponding to his two-dimensional point set *D_i_*, into *K_i_* disjoint intervals through SVD algorithm, then obtains *K_i_* rectangular data partition *B_i_*_,*k*_ presented in Figure 6, i.e., *D_i_* = <*B_i_*_,1_, *B_i_*_,2_, …, $B_{i,K_{i}}$>. Obviously, the rectangular partition can be uniquely identified by its lower-left (*LL*) and upper-right (*UR*) corners.
(3) *O_i_* randomly select a number *r*_*o_i_* and encrypt *K_i_* data partition above through the using of *Key_O_i_* and *r*_*o_i_*, then obtains *K_i_* data items in the format shown in Figure 7. The process of encryption is described in Algorithm 5.
  **Algorithm**
**5.** BlockEncryption.Input：*Key_O_i_*, *r*_*o_i_*, *K_i_* data partition.Output：*K_i_* data items in the format shown in Figure 7.
(a)**WHILE**(1 ≤ *k* ≤ *K_i_*)
(b)Encrypt the *LL* and *UR* of *B_i_*_,_*_k_* to get $E_{Key\_O_{i}，r\_o_{i}}(x_{LL})$, $E_{Key\_O_{i},r\_o_{i}}(y_{LL})$, $E_{Key\_O_{i},r\_o_{i}}(x_{UR})$, $E_{Key\_O_{i},r\_o_{i}}(y_{UR})$(c)Encrypt the points contained within the scope of *B_i_*_,*j*_ to get ${\left\{E_{Key\_O_{i},r\_o_{i}}(t)\right\}}_{t\in B_{i,k}}$, where $E_{Key\_O_{i},r\_o_{i}}(t)=\langle E_{Key\_O_{i},r\_o_{i}}(x_{t}),E_{Key\_O_{i},r\_o_{i}}(y_{t})\rangle$(d)construct the data item in the format shown in Figure 7(e)**END WHILE**
(4) *O_i_* uploads the data items generated in Step 3 to the CS and send *r_o_i_* to the PS.

### 5.3. Access Control

In our method, the users have the capacity to access the encrypted data on the CS via the PS, which are uploaded by the Owners. In the real scenario, however, not all of the data can be visited by all users, only the user who was authenticated by the data owner can access the uploaded data. Hence the access policy in our system is necessary. In this paper, we use ABE [30] to achieve access control in which the data owner has the right to set access policy, so it is suitable for the data-sharing of crowd sensing networks.

For example, in VANET, a data collector as the data owner will outsource their data to the cloud, but these data are only expected to open to the owners of the A-region and the B-car. Naturally, he informs the management department as the TA of the access condition. Before owners visiting the data stored in cloud through a proxy server of the manufacturer, the proxy needs to send the owner’s attributes (area, automaker, etc.) to management department for the permission to the specific dataset. Owners cannot visit the data in the cloud via the proxy until the condition is met.

**Figure 8:**
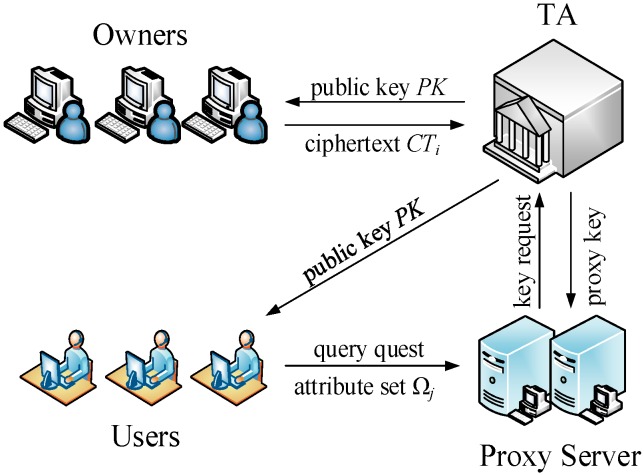
The SVD method for *K* = 4.

**Figure 9:**
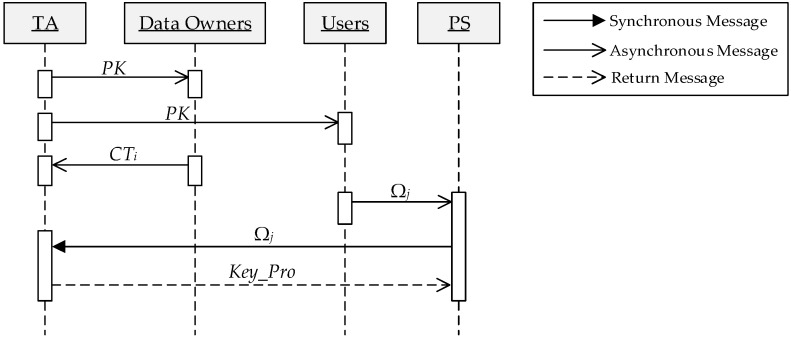
The protocol sequence diagram.

The framework of access control is shown in Figure 8. All data owners upload their ciphers that contain access policy to TA. After receiving a query request from the User, PS sends the user’s attributes to TA to be verified and obtains the proxy key. Once being verified by TA, PS can obtain the proxy key and perform the next phase of the query over the corresponding dataset in the cloud. The protocol sequence diagram in access control phase is shown in Figure 9. Specific processes are as follows:

(1) TA generates the public key *PK* and the master key *MK* used in ABE and publishes the *PK*.
(2) The data owner *O_i_* with a access policy *AP_i_*, *PK* and *Key_O_i_* computes ciphertext *CT_i_* and sends it to TA.
(3) The data user *U_j_* sends his own attributes Ω*_j_* to PS.
(4) PS sends Ω*_j_* to TA for requesting a proxy key.
(5) TA with Ω*_j_* and *MK* outputs the user’s attribute private key *ASK*[Ω*_j_*] and inserts *Key_U_j_* into the header of the proxy key chain.
(6) TA takes *CT_i_* and *ASK*[Ω*_j_*] as input, achieves a decryption to get *Key_O_i_* and inserts it into *Key_Pro_j_* only if Ω*_j_* fully meets the access policy, otherwise output ⊥, i.e., this user does not have permission to access the data of *O_i_*. This step is repeated until all of the dataset were visited, as a result, generates a complete proxy key chain *Key_Pro_j_*.
(7) TA sends *Key_Pro_j_* to PS.

### 5.4. Result Query

The data user *U_j_* randomly chooses a parameter *r*_*u_j_*, encrypts the query point *Q* with *Key*_*U_j_* and *r*_*u_j_*, then sends the encrypted query and *r*_*u_j_* to PS. Next, PS transfers the encrypted query to CS and then destroys it locally (This is a reasonable action, because a person would not keep all secret shares locally for the data security). Through the access control process described in Section 5.3, the proxy gets the key chain *Key_Pro_j_*, i.e., *U_j_* can visit the data in the cloud via a proxy server. Suppose *U_j_* can get a permission for *w* dataset, then *Key_Pro_j_* = {*Key_U_j_*, *Key_O*_1_, …, *Key_O_w_*}. Let $E_{Key\_O,r\_o}(D)$ = {$E_{Key\_O_{1},r\_o_{1}}(D_{1})$,$E_{Key\_O_{2},r\_o_{2}}(D_{2})$, …, $E_{Key\_O_{w},r\_o_{w}}(D_{w})$} denote *w* corresponding dataset in the cloud, where $E_{Key\_O_{i},r\_o_{i}}(D_{i})$ is composed of *K_i_* data items shown in Figure 7.

Firstly, the PS randomly selects a key *sk_q_* and a parameter *r_q_* for a query. The PS and the CS view *sk_q_* and *r_q_* as a normalized key and a normalized parameter for this query, respectively. The encrypted data in the CS were given normalized treatment by DataNorm algorithm. Then the PS and the CS find a block that contains the query point by the SecComp algorithm, i.e., find a block *B*, making *x_Q_* > *x_LL_*, *y_Q_* > *y_LL_*, *x_UR_* > *x_Q_* and *y_UR_* > *y_Q_*. Repeat the above operation over dataset of size *w* until output an encrypted result point to the *U_j_*. At last *U_j_* decrypts the ciphertext to obtain a nearest neighbor. The process of SNN query is described formally in Algorithm 6.

**Algorithm 6.** SNN-SAMOMU.Require: CS has *E_Key_O_*,*r*_*o*(*D*) and $E_{Key\_U_{j},r\_u_{j}}(Q)$    PS has *Key_Pro_j_*, *sk_q_* and *r_q_*    *U_j_* has *Key_U_j_* and *r_u_j_*.(1)PS and CS: **FOR** 1 ≤ *i* ≤ *w*
(a)CS get $E_{sk_{q},r_{q}}(D)$ and $E_{sk_{q},r_{q}}(Q)$ by DataNorm algorithm(b)get the block *B* where the nearest neighbor locate by SecComp algorithm(c)**WHILE** (*t_k_*∈*B*)$E_{sk_{q},r_{q}}(d_{k})\leftarrow \mathrm{Sec}\mathrm{Dist}(E_{sk_{q},r_{q}}(Q),E_{sk_{q},r_{q}}(t_{k}))$**END WHILE**(d)*Min_i_* ← ${\mathrm{Sec}\mathrm{Min}}_{K_{i}}(E_{sk_{q},r_{q}}(d_{1}),\ldots ,E_{sk_{q},r_{q}}(d_{K_{i}}))$(e)get δ where *Min_i_* == $E_{sk_{q},r_{q}}(d_{\delta })$(f)*Res_i_* = $E_{sk_{q},r_{q}}(t_{\delta })$ and *Min_i_’* =$E_{sk_{q},r_{q}}(Min_{i})$(2)PS and CS:
(a)*Min*’ ← SecMin*_w_*(*Min*_1_’, *Min*_2_’, …, *Min_w_*’)(b)get ξ where *Min’* == $Min_{\xi }$’(c)get Res = DataNorm($Res_{\xi }$,{*sk_q_*,*r_q_*},{*Key_U_j_*,*r*_*u_j_*})(3)CS:
(a)Send *Res* to *U_j_*(4)*U_j_*:
(a)NN ← $D_{Key\_U_{j},r\_u_{j}}(Res)$

### 5.5. Optimization

Our scheme runs on the top of encrypted data for the SNN query, whereas it does introduce inefficiency. Now we discuss two strategies to boost the efficiency: offline computation and pipeline execution.

In our protocols, the actual online computation costs with an offline phase can be much less than their costs without an offline phase. For example, consider the DataNorm primitive described in Algorithm 1. During the execution of DataNorm, *P*_1_ has to compute the encrypted value *s* = ($m_{3}g^{a_{3}}$ mod *n*)^−^^1^, where *m*_3_, *a*_3_ and *r*_3_ are random numbers in *Z_N_*. However, since these numbers are integers chosen by *P*_1_ at random, the computation of *s* is independent of any specific factor of DataNorm. That is, *P*_1_ can precompute the value of s during the offline phase, thus reducing its online computation time. In a similar manner, *P*_1_ and *P*_2_ can precompute certain intermediate values in the protocols.

We are able to further reduce the online execution time by adopting the technique of pipeline execution. Take the execution of SecMin*_k_* for instance, *P*_1_ and *P*_2_ would like to process SecComp(*d*_1_, *d*_2_) and SecComp(*d*_3_, *d*_4_). Here the execution of SecComp(*d*_3_, *d*_4_) does not have to wait for the end of SecComp(*d*_1_, *d*_2_). Instead, they can be executed synchronously. We expect that we could further save at least one-third of the online execution time in the long run when we have a lot of SecComp operations to perform. Likewise, we could pipeline the SNN-SAMOMU protocol to save much time.

## 6. Security Analysis and Performance Evaluation

In this section, we analyze security properties of the proposed scheme, and show that it achieves the defined security design goals. We then provide the performance evaluation on our scheme.

### 6.1. Security Analysis

#### 6.1.1. Data Confidentiality

In our method, Owners segment the data set through SVD algorithm [15] and encrypt their own data and index by the encryption scheme described in Section 4.1. The data confidentiality in the above process is ensured by the following theorems:

**Theorem 1** ([15])**.** *If E is a secure encryption scheme in a standard security model M, then SVD method is as security as E in the same model M with respect to a single query. For the details of the proof, refer to [15].*

**Theorem 2** ([31])**.** *The encryption scheme described in Section 4.1 can be against chosen plaintext attack (CPA) threat. For the details of the proof, refer to [31].*

Now we analyze that our method can resist adversary *Adv* which achieve data confidentiality. If *Adv* eavesdrop the transmission link between Owners and CS, the encrypted values $E_{Key\_O_{i},r\_o_{i}}(D_{i})$ are got by *Adv*. Moreover, all the intermediate values transmitted between PS and CS may also be eavesdroped by *Adv*. Because all these data are transmitted in encrypted form and are randomized by the parameters *r*_*o_i_* or other random numbers involved in the protocols, it is impossible for *Adv* to decrypt the ciphertext and intermediate values without knowing the Owners’ keys or parameters.

Next, suppose *Adv* compromises a specific Owner *O_z_* and CS simultaneously, to get all encrypted data stored in CS, *O_z_*’s secret key *Key*_*O_z_* and parameters *r*_*o_z_*. However, *Adv* cannot recover the plaintext of other Owners except for *O_z_*. Because all Owners encrypted the data with their own secret keys and random parameters. In addition, all the intermediate values in CS are encrypted with *sk_q_* or randomized by *r_q_* during each query. In all, *Adv* cannot know any assistance to decrypt the encrypted data, i.e., the data confidentiality, defined in Section 3.2, was satisfied.

#### 6.1.2. Query Privacy

Here, we analyze that our method can resist adversary *Adv* which achieve query privacy. If *Adv* eavesdrop the transmission from the User *U_z_*, the encrypted query $E_{Key\_U_{z},r\_u_{z}}(Q)$ are got by *Adv*. However, *Adv* cannot recover the query point without knowing the *U_z_*’s secret keys *Key_U_z_* and the parameter *r*_*u_z_*. Next, suppose *Adv* compromises CS, some Users and Owners simultaneously, to get some Users’ and Owners’ secret keys and parameters and intermediate values during a query. It is also too hard to get any information that can reveal the actual query point. Because all computations are implemented on encrypted data and all the intermediate values that contain the query point are randomized in the query protocol. In conclusion, the query privacy defined in Section 3.2 was satisfied in our method.

### 6.2. Performance Evaluation

We developed a Java prototype that implements our method (SNN-SAMOMU). More specifically, we make use of: (a) an Alibaba Elastic Compute Service (ECS) instance with quad-core Intel Haswell CPU at 2.50 GHz, 16 GB RAM as cloud server; (b) a desktop with an Intel(R) 3.30 GHz CPU and 16 GB RAM running Windows 7 as proxy server; and (c) a laptop running Windows 7 with 2.80 GHz CPU and 4 GB RAM as client (data user and owner). The maximum communication bandwidth between the cloud server and the proxy server is set to 10 Mbps, while that between the client and the servers is set to 1 Mbps.

**Figure 10:**
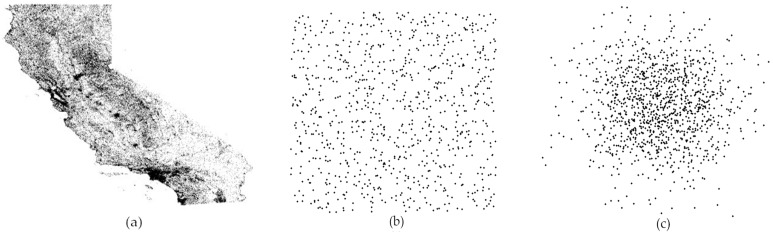
Different datasets: (**a**) real-world; (**b**) uniform distribution; and (**c**) standard normal distribution.

To make a comprehensive performance evaluation, our experiments are implemented on three different datasets (as shown in Figure 10): (a) a real-world dataset from California’s Points of Interest [32] which contains 104,770 location records; (b) a synthetic dataset following uniform distribution; and (c) a synthetic dataset following standard normal distribution. We test our scheme over these datasets with different scales of data size (from 20,000 to 100,000). At least 30 random NN queries are selected and evaluated with each scale. In addition, we used the Qhull library to find the Voronoi diagram for the dataset *D* and used SVD method to ensure each rectangular partition has roughly 1000 points. For encryption scheme, we used 1024-bits keys. Table 2 presents the specific parameter settings in our experiment.

The main performance metrics used to evaluate the proposed scheme are data processing time at the data owner, query response time and communication cost at the user. We compare our scheme with two existing schemes, the SVD-SNN method [15] and the VD-1NN method [18].

#### 6.2.1. Data Processing Time at the Data Owner

**Figure 11:**
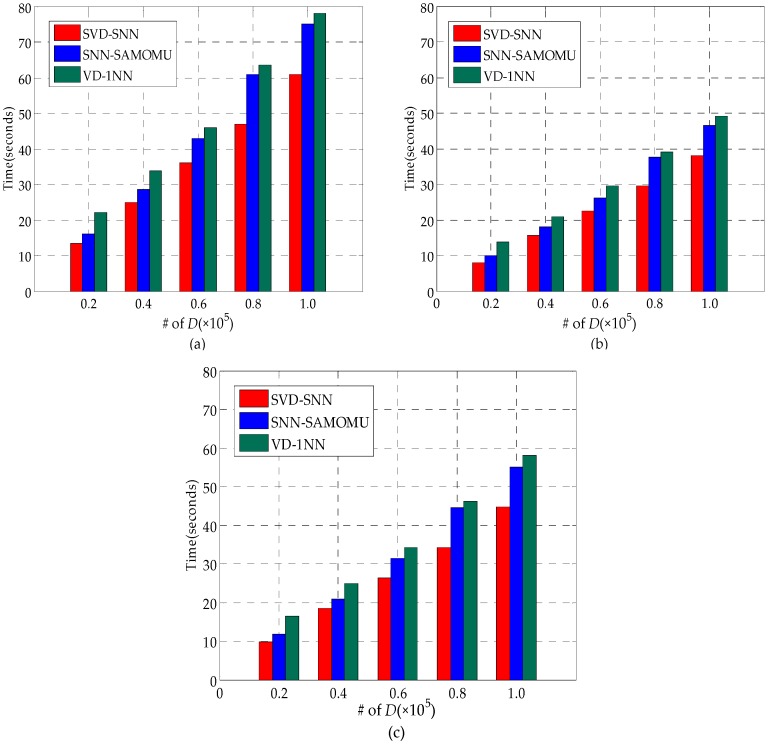
Data Processing Time at the Data Owner: (**a**) real-world; (**b**) uniform distribution; and (**c**) standard normal distribution.

In the procedure of data pretreatment, there are two major steps for the data owner: performing SVD algorithm and encrypting data. As we can observe from Figure 11, with an increase of the data size, the data processing time increases. It is extremely efficient when the data size is small, but relatively inefficient when the number of records in the dataset reaches 100,000. For instance, it only requires 13.5 s on the real-world dataset (Figure 11a) with 20,000 records, while the data processing time is about 80 s with 100,000 records. However, this is only a one-time cost. Besides, spending more time to build an index in order to optimize query time is the essential methodology.

In Figure 11, the data processing time of our scheme is somewhere between SVD-SNN and VD-1NN because the owner in the SVD-SNN encrypts the data with AES, which is an efficient cryptographic primitive, while the owner in VD-1NN has to compute many auxiliary parameters beside of encrypting all the points. Another observation is that these three schemes exhibit the best performance on the uniform dataset (Figure 11c), whereas they show the worst performance on the real-world dataset (Figure 11a). This is because uneven density of the real-world dataset causes SVD algorithm to be highly inefficient.

#### 6.2.2. Query Response Time

**Figure 12:**
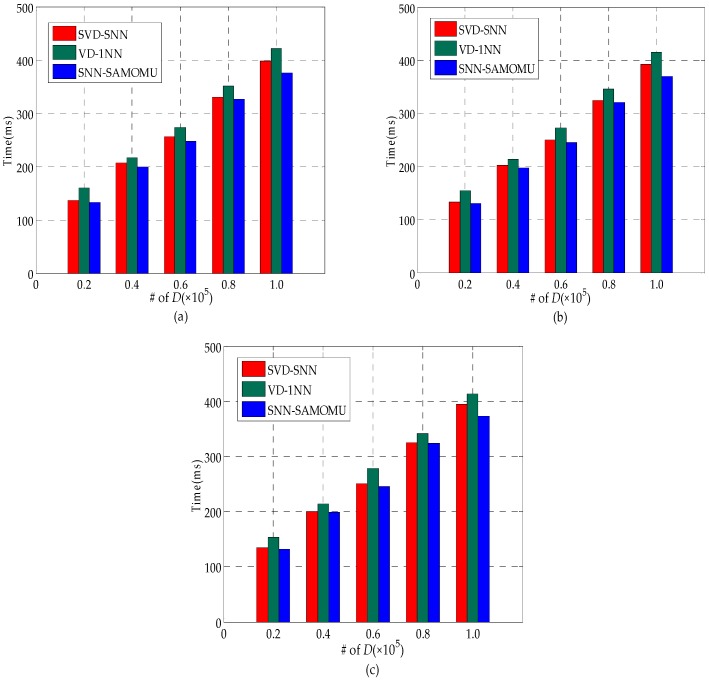
Query response time: (**a**) real-world; (**b**) uniform distribution; and (**c**) standard normal distribution.

**Figure 13:**
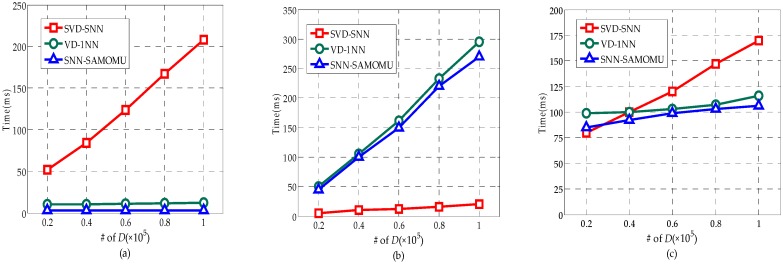
Response time break on the real-world dataset: (**a**) User CPU time; (**b**) Server CPU time; and (**c**) communication time.

The main performance metrics used to evaluate the proposed technique are query response time. This indicator measures the duration from the time the query is issued until the results are received at the end-user. It includes the computation time at the proxy server, the cloud server and the client, as well as the communication time,å which makes up a considerable percentage of total time. Figure 12 shows the query response time for all considered methods under different datasets. As we can see in Figure 12, different distributions have limited effect on query response time for these methods, since all values are treated in a similar way in encrypted form. Furthermore, we can find our scheme is slightly better than others. In order to show the superiority of our method, Figure 13 provides a breakdown of the response time into the server CPU time, the end-user CPU time and the communication time on the real-world dataset. Note that the server CPU time consists of the proxy server CPU time and the cloud server CPU time.

Figure 13a shows the end-user CPU time in our method is significantly less than the SVD-SNN method. It is because the users in the SVD-SNN method have to decrypt the partition contains a lot of candidate points rather than a result point. Figure 13a also shows our method is slightly better than VD-1NN method about the end-user CPU time. Another important observation is that the end-user CPU time in our method remains the same with growth of the database scale because the encryption and decryption operation need to be done only once for each query, regardless of data size. More particularly, the end-user in our method only requires a total of 6 ms on average during the SNN query. These are desirable features for MOMU model, as end-users are lightweight devices with limited computation capabilities.

In Figure 13b, the lower server CPU time for SVD-SNN is due to the fact that it encrypts the data by AES for increased query efficiency. However, this way decreases the data availability dramatically. The server CPU time in our method is slightly less than the VD-1NN and linearly related to the size of the dataset. Figure 13c shows our method has the best performance for the communication time, which has benefited from the fact that the interactive query time between the proxy and cloud server had the highest proportion of the total time while the interactive time between the user and the server is the most time-consuming in other methods.

#### 6.2.3. Communication Cost at the User

**Figure 14:**
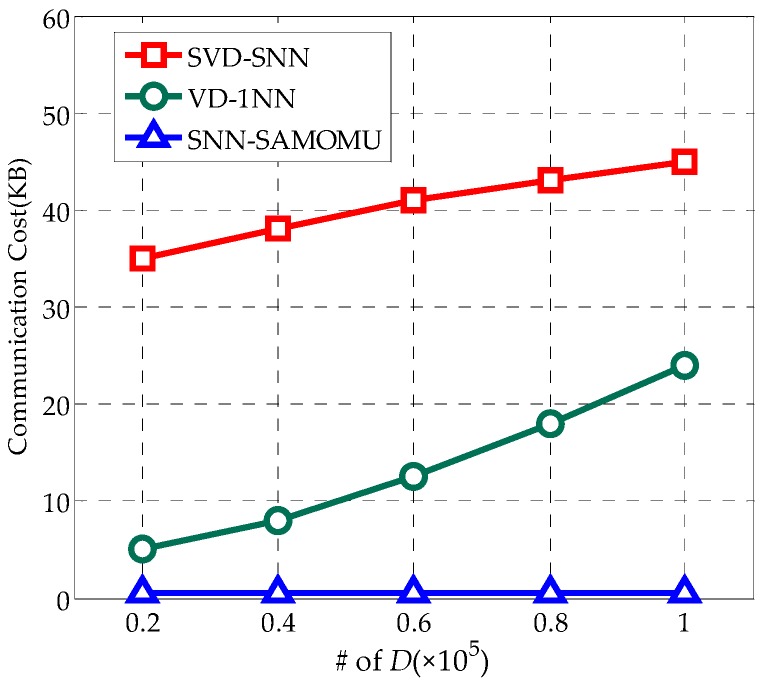
Communication Cost at the user.

In the experiment, the communication cost is the amount of data transferred between the servers and the user. In Figure 14, it is obvious that the cost in our method is almost negligible while the amount of communication grows with the size of the dataset *D* in others. This is due to the fact that we use the proxy server to share the hard work for the end-user. However, the users in SVD-SNN are required to receive a large number of indexes and data partition. In VD-1NN, as the result of a mutable order-preserving encryption, the users have to interact with the cloud frequently.

## 7. Conclusions

In this paper, we focus on the secure nearest neighbor (SNN) problem on crowd-sensing location data. The previous SNN techniques generally rely on the Single Owner and Multi Users (SOMU) model, which only contains a single trusted data owner. However, the previous big data system structure has changed because of the crowd-sensing data, i.e., the security and performance requirements have changed. Given all this, we proposed a SNN query scheme based on the SAMOUMU model, which is constructed by the protocols of secure two-party computation and SVD algorithm. We showed a theoretical analysis that our scheme can protect the data confidentiality and query privacy. Finally, extensive experimental evaluations are presented to show that our scheme is applicable to crowd-sensing data and significantly lower the users’ cost. As a future work, we will extend our method to *k* nearest neighbors and further reduce the server’s cost.

## Figures and Tables

**Table 1:** Requirements and Key Techniques under SAMOMU model.

Requirements	Key Techniques
Data confidentiality and query privacy	SVD method and the encryption based on secret-sharing
Reduce the end-users’ cost	secure two-party computation protocols
Access Control	attribute-based encryption

**Table 2:** Parameter Settings.

Parameter	Values
Maximum communication bandwidth between the cloud server and the proxy server	10 Mbps
Maximum communication bandwidth between the client and the servers	1 Mbps
Size of the dataset *D*(×10^5^)	0.2, 0.4, 0.6, 0.8, 1.0
Size of each rectangular partition	1000
Size of the keys	1024 bits
